# On Wor(l)ds and Pandemics

**DOI:** 10.1007/s10912-024-09873-x

**Published:** 2024-07-22

**Authors:** Jorge J. Locane

**Affiliations:** University of Oslo, ILOS, Oslo, Norway

**Keywords:** Pandemic, World, COVID-19, Smallpox, Latin America

## Abstract

The spread of the coronavirus SARS-CoV-2 has stimulated eschatological speculation. To the environmentalist and liberal diagnostician that had already been warning about the Anthropocene and the breakdown of post-Cold War global harmony, an alarm has now been added that in its worst prognosis estimates that, in 2020, we only started witnessing the beginning of a staggered health debacle. The idea of the world, as conceptual support for an imaginary community with global reach, has become a crisis. The world, an object often invoked by theoretical speculation over the last 30 years, has been now decreed finished. However, infectious diseases, in their epidemic and pandemic form, have devastated different societies at different times. This paper parallels two historical scenarios and a series of texts dealing with contagious diseases to shed light on the idea of (the end of) the world. The analysis centres on documents that bear witness to the importation of smallpox and other diseases into America and its spread during the European invasion and colonization. By recovering the concept of Pachakuti, a radical turnaround that can be understood as “end of one world”, this paper shows that the chronicles reporting on the outbreaks of smallpox in America document a material end of the world for subjects who were not protagonists of history. The current end of the world is, on the contrary, that which would correspond to the protagonist of our phase of globalization and, eventually, to *his* world—which makes it more resonant and absolute.

Published right before the COVID-19 pandemic outbreak, Karen Thornber’s groundbreaking and comprehensive book, *Global Healing* (2020), has shed some light on the intertwining between illness and globality. Although the disease affects humans and, in certain cases, also non-humans alike, cure and death are goods globally unequally distributed. This means that vulnerability should always be considered from an intersectional approach, including here the variable of the physical location in an uneven world-system. Certainly, Thornber’s book has challenged the medical humanities by introducing a perspective that, by addressing literary sources from all over the world, encourages us to go beyond the individual case or the phenomenon located in the subject or even the isolated community. However, Thornber resorts to an ambitious global or comparative perspective in her methodological approach. The fact of considering the disease in its global and cross-cultural dimension seems to emerge a posteriori, as a side effect of her readings: “The more I read, the more patterns and connections became visible, and the clearer it became how each story fitted into a broader global context, even though local and regional circumstances were often very different” (2020, 8). Even so, Thornber’s contribution may have anticipated a turn that will likely occur in the medical humanities in the aftermath of the COVID-19 pandemic. The task of examining articulations between the individualized manifestation of illness and literature has been carried out, since Susan Sontag’s seminal book (1978), by a prolific interdisciplinary perspective that straddles medicine and literary studies^1^. Thornber’s book, even considering its comparative and global perspective, can still be located within this tradition insofar as it keeps the focus on the individual subject affected by a disease, his or her suffering and that of his or her family environment.

However, as the SARS-CoV-2 virus spread around the world, what we human beings experienced is that none of us is a monad. If there is one thing that modern Western subjectivity may have discovered in the most recent years, it is that, for better or worse, as human beings, we are a collective unit that transcends not only individual bodies but also national constructions and even the geophysical boundaries set by seas or mountains. The approach of this paper is consequently based on a communal definition of disease: it focuses on contagious diseases, how they spread between different regions of the world and affect populations or societies as a whole.

In addition to illness,^2^ thought of in terms of its power to spread, and literature, this contribution will address the element “world” and introduce a Latin America-focused approach. More specifically, the aim of this research paper consists in discussing the spread of contagious diseases and the associated perception of the “end of the world” by contrasting different cases and theoretical assumptions. For the purposes of the following arguments, the world is to be understood from different angles and in its semantic ambiguity: as an objective material expression, but also as a fundamentally liberal and rhetorical project for overcoming ethnic and national boundaries. The world, just to begin with, may be the sum of all the human and non-human configurations that inhabit the earth, but, in some cases, it is also made of *words* insofar as it is the effect of a normative—perhaps too voluntarist and insistent—theoretical enunciation. I will delve into some of the complexities related to this in the next pages.

Two quotations can serve as a starting point to reflect on communicable diseases and the world. The first reads:

If a killing type of virus strain should suddenly arise by mutation... It could, because of the rapid transportation in which we indulge nowadays, be carried to the far corners of the earth and cause the deaths of millions of people – W. M. Stanley, in *Chemical and Engineering News*, December 22nd, 1947. (Stewart 1979, 9)

These words introduce Part I of *Earth Abides* which may be defined as a dystopian science fiction novel. The world it portrays is one after the near-total extinction of mankind due to a lethal virus. Although the epigraph may suggest otherwise, the author, George R. Stewart, did not publish *Earth Abides* in 2021 but in 1949. The quotation is by the virologist and Nobel laureate in chemistry Wendell Meredith Stanley and deserves to be highlighted because of its indisputable topicality and because it allows us to deploy all sorts of arguments about the visionary power of literature. Speculative fiction announces possible worlds and, in some cases, seems not to fail. By predicting a future state, speculative fiction can change its character in the course of history and come to be perceived as realistic, that is, as a non-fiction document. This is precisely what, in relation to *Earth Abides*, Tawhida Akhter’s paper (2022) allows us to think about, since it draws parallels between the context of the COVID-19 pandemic and Stewart’s post-apocalyptic scenario to the point of concluding that “The COVID-19 pandemic has halted the whole world and has infected every corner of the world. This pandemic is the deadliest pandemic in human history-wiped out all the fields of humanity, as in *Earth Abides*” (2022, 148). Leaving aside the exaggerations—which in some ways will be dealt with later in this paper as the fruit of ethnocentric bias—we can always discuss how and to what extent the speculative genre may become realistic. Other works of fiction that seem to have updated their status in the context of the COVID-19 pandemic include films such as *Contagion* (2011) and the Japanese movie *Virus: Day of Resurrection* (1980) (Brito-Alvarado and Martínez Bonilla 2021). Many viewers have watched these films not in the context of their original release but after 2020 and not as films that imagine scenarios alien to their everyday reality but more or less verifiable in their historical experience. Speculative fiction and realism can therefore be conceived as contingent variables, subject to the vicissitudes of history.

The second quote is as follows:

a great plague broke out here in Tenochtitlan. It began to spread during the thirteenth month and lasted for seventy days, striking everywhere in the city and killing a vast number of our people. Sores erupted on our faces, our breasts, our bellies; we were covered with agonizing sores from head to foot.

The illness was so dreadful that no one could walk or move. The sick were so utterly helpless that they could only lie on their beds like corpses, unable to move their limbs or even their heads. They could not lie face down or roll from one side to the other. If they did move their bodies, they screamed with pain. A great many died from this plague, and many others died of hunger. They could not get up to search for food, and everyone else was too sick to care for them, so they starved to death in their beds.

Some people came down with a milder form of the disease; they suffered less than the others and made a good recovery. But they could not escape entirely. Their looks were ravaged, for wherever a sore broke out, it gouged an ugly pockmark in the skin. And a few of the survivors were left completely blind. (León-Portilla 2006, 92-93)

Beyond the difference in approach, this too is a story about the spread of a disease. One might think that the virus announced in the first quote, thanks to “rapid transport”, reaches “a distant corner of the earth” and wipes out the local population. The quote comes from the book *The Broken Spears* (in Spanish: *Visión de los vencidos*, 1959) which is one of the many accounts written by Spanish or Mestizo *cronistas* about the arrival of the conquistadors in America and, with them, devastating epidemics. *The Broken Spears* tells the story of the invasion and fall of Tenochtitlan to the Spanish in 1521, after the penultimate Mexica *tlatoani*, Cuitláhuac, died of smallpox. Shortly before his death, he had led the fight against Hernán Cortés and his troops and succeeded in driving them out of Tenochtitlan. The texts that make up the book come from different codices written in Nahuatl and translated by José León-Portilla into Spanish for its first publication in 1959.

Unlike the first reference, there is no speculative fiction here. It is a non-fiction document, a testimony, which projects descriptively toward the immediate past. In fact, *The Broken Spears* was reprinted in 1969 by the Cuban publisher Casa de las Américas and thus incorporated into the canon of Latin American testimonial literature (Curiel 2009). When comparing the verb tenses of both quotations, it can be seen that those of the first quotation refer to a potential future while those of the last one to the past, to a closed and irreversible historical fact. In the first case, the end of the world, caused by the spread of the virus referred to in the quotation, is something that could happen in a possible future. In the second, on the contrary, the tragedy is conclusive, it has already happened: the end of the world here is therefore an episode of the past. In the following, to deepen the arguments, we will enter into two scenarios based on the two quotations.

## Scenario 1

The first scenario is set temporally in the present. Many fictions, such as the aforementioned film *Contagion* (2011), the novel *Station Eleven* (2014) by the Canadian Emily St. John Mandel, or *Los que duermen en el polvo* (2017), by the Argentinean Horacio Convertini, have been predicting what we can call the dystopia of our generation. *Station Eleven*, in fact, found a new echo in the context of the COVID-19 pandemic and was recreated as a miniseries by HBO. In all these cases, a virus or a plague spread through the world and ended up wiping out what we know as human civilization. It is worth noting, however, that the collapse never becomes as radical as extinction since there are always human survivors who take on the responsibility of telling the story and refounding society. These narratives are part of a wider body of discourse concerning a generalized and irreversible crisis. In a way, the lethal virus spreading around the world appears in world history as the end point of the Anthropocene, and thus as the closure of an escalating crisis, centred on the human as agent. Aronsson and Holm (2022), for example, argue that anthropogenic phenomena such as deforestation or climate change have led to new interspecies contacts that favour an unusual spread of viruses and bacteria. So, as Simon Dein (2020, 10) suggests, the sum of critical indicators in different spheres has fuelled an apocalyptic imagination in the context of the COVID-19 pandemic, both in its religious and secular sense.

Every society has its eschatological narrative, from the Christian apocalypse to the Nordic Ragnarök. It happens, however, that in our current dystopia, discourses that had been rivals seem to have aligned. The statements, for example, of the Earth sciences and climatology—which can be considered translators of nature’s messages—coincide with the postulates of speculative fiction as well as with those of scholars such as Donna Haraway or Bruno Latour. The spread of a *real* virus throughout the world, as announced by the Nobel Prize winner Stanley in 1947, has added, moreover, empirical evidence permanently recreated by the media in all countries. Thus, not only the discourses but also the times of the experience of the end have been synchronized. The virus, present through the media and social networks in the lives of everyone everywhere on the planet, has reaffirmed the idea that it is possible for the world to end and for it to end, in principle, for everyone, at least for human beings.

To the dismantling of welfare states, environmental disaster—from the acidification of the seas to the devastation of forests and global warming—and massive and forced human displacement, among other facts, has now been added the threat of a “global” virus. As Mike Davis has examined (2022), this virus, moreover, had already been anticipated in the twenty-first century by “not-so-aggressive” viruses or “not-so-global” pandemics such as H1N1 influenza and avian flu. So, even when “Throughout history pandemics and apocalyptic narratives have run closely together” (Dein 2020, 6), the perception seems to be that today the end of the world is more inevitable or material than ever.

The word world, however, has several semantic complexities. I will examine some of them in more detail at the end of this contribution. Although the COVID-19 pandemic has led to a widespread perception of catastrophe,^3^ I want to stress here that it accentuated the *crisis of the world* in two of its various senses: on the one hand, in terms of its biological dimension—at least as far as human beings are concerned—, since we now take it for granted that a lethal virus can wipe out human life and that this does not only happen in novels or films. On the other hand, it affects the world as the horizon of the liberal project, both in its mercantile expression, that is, in what refers to the world as a global market without borders, and in the normative formulations of radical humanism or—as Walter Mignolo would say (2012)—of the “honest liberals”. Due to the pandemic, national borders have been reconstituted, and the liberal utopia of a non-interventionist state as well as the free movement of people has been revealed as blatant fantasies.^4^ Since the threat always manifests itself as coming from outside or from otherness, due to the pandemic, the status of “foreigner” has become more in force than ever. Previously, the condition was present but concealed by state political correctness. Where and how do the many people who could not prove official residency in the cities of the Global North, or elsewhere, have been living during the COVID-19 pandemic? At the height of the pandemic, vaccines were only available to documented residents. Norway is a country where undocumented residence is virtually non-existent and where the prophylactic policy to prevent the spread of the SARS-CoV-2 virus consisted mainly of severely restricting access to the country. On 27 January 2021, *Aftenposten*, the country’s largest circulation newspaper, reported that 50% of those infected by the coronavirus were foreigners: “Nå er over halvparten av koronapasientene i Norge født i utlandet” (Johansen and Stokke 2021). The note does not report that in the main cities, where the higher population density favours the transmission of communicable diseases, the immigrant population reaches 30% and that due to their jobs and the need to maintain contact with the outside world, these residents were perhaps the most exposed to contagion. What is remarkable, however, is that in the face of a virus that seemed to affect everyone without distinction, the press has emphasized the “foreigner” variable. Once again, the disease belongs to the other. In this case, to the one who comes from “outside”, just as HIV was considered a virus of “deviant” sectors of society.

I would like to close this scenario with a brief reflection on the world made of words, i.e. on “wor(l)ds”. This term refers to a normative definition of the world advocated by a theoretical cosmopolitanism that has its more conspicuous precedent in texts by Immanuel Kant, such as “Idea for a Universal History with a Cosmopolitan Purpose” (1784), and today has, among others, prominent promoters such as Pheng Cheah and Mariano Siskind. For this strand of critical thought, the world does not really exist. Rather, it is a desiderative statement to which fiction can and should contribute. “Since one cannot *see* the universe, the world, or humanity”, Pheng Cheah writes, “the cosmopolitan optic is not one of perceptual experience but of the imagination” (2008, 26). The historical experience of widespread debacle has recently led Siskind to write of “the end of the world”. According to Siskind, “World named the modern and modernist symbolic structure that supported humanist discourses of universal emancipation through global connections, translations, interactions, displacements and exchanges; ‘world’ as the symbolic realm where demands of justice, emancipation and universal inclusion (whether political, cultural and/or aesthetic) were meant to be actualized” (2019, 207). This utopia of universal harmony seems to have failed. There are no longer any conditions of possibility that allow us to imagine a world. The world, in any case, is not interconnected, and justice for all human—and I would add non-human—entities is far from being achieved. We are witnessing, therefore, “the symbolic closure of the horizon of universal justice and emancipation that had defined the modern/modernist relationship between cosmopolitan politics and culture” (Siskind 2019, 209). What seems to be exhausted is the possibility of creating a common world capable of overcoming the contradictions of nationalism and ethnocentrism. This scenario, which corresponds to the twenty-first century, seems to be marked by this *Zeitgeist*. As Dein points out, “Apocalypse has now become a popular cultural trope in fiction, film and popular discourse” (2021, 10), but also the theoretical speculation that had placed its expectations in the production of the world has lately adopted an apocalyptic tone.

## Scenario 2

This second scenario has more than 500 years of history. It begins, to be exact, on 12 October 1492, when Europeans set foot on American soil, thus inaugurating Western modernity. The passage from *The Broken Spears* quoted at the beginning of this paper is one of many possible examples of the biological imbalance produced by colonization. Along with the brutality of arms and forced evangelization, the invaders—without exactly knowing it—used contagious diseases to subdue the vernacular populations. One particularly effective biological weapon was smallpox, which—as documented in studies by Ashburn (1947), Cook, Crosby, Mann, and Stannard based on information from the chronicles of the conquest—spread with the Spaniards from the Antilles to what is now Mexico and then southwards to Tawantinsuyu.^5^

The images provided by the chronicles of the time are truly *apocalyptic*. In assessing the contingencies of the invasion of America, it is inevitable to consider that the vernacular peoples had not been exposed to the diseases brought by the Spaniards, nor did they even have prior information about the existence of the invading culture. For the natives, the discovery of these people who, moreover, came on horses and carried firearms—among many other elements unknown in America—occurred at the same time as the arrival of great plagues. Thus, the invaders were considered unearthly entities that came to their domains to impose devastation by means of unintelligible resources.

It should be noted here that Europeans were, as a rule, immunized. They had already obtained what we craved during the most pressing moments of the COVID-19 pandemic: herd immunity. In his study *American Holocaust* (1992), David Stannard highlights the marked differences in the living conditions of Europeans and pre-Columbian populations in the Americas. Hunger, misery, and disease were distinctive features of Europe, while climatic conditions and some social development had led to relative prosperity in the Americas. Stannard argues that “unlike the island natives the European invaders and their forebears had lived with epidemic pestilence for ages. Their lungs were damaged from it, their faces scarred with pocks, but accumulations of disease exposure allowed them now to weather much. So they carried infections with them everywhere they went—burdensome, but rarely fatal, except to the natives that they met” (1992, 69).^6^ Contagious diseases, therefore, spread rapidly among the indigenous people, but did not affect Europeans. According to Alfred Crosby,

Where smallpox has been endemic, it has been a steady, dependable killer, taking every year from 3 to 10 percent of those who die. Where it has struck isolated groups, the death rate has been awesome. Analysis of figures for some twenty outbreaks shows that the case mortality among an unvaccinated population is about 30 percent. Presumably, in people who have had no contact whatever with smallpox, the disease will infect nearly every single individual it touches. (2003, 44)

American societies belong to the latter category. Smallpox arrived with a carrier and quickly began to circulate among the natives until it became a lethal epidemic. Moreover, since in some regions of the continent the urban infrastructure was not negligible and the demographic development significant—“because of the rapid transportation”, one could say playing on Stanley’s quote—the contagion was extremely agile (Mann 2005, 96–97). Smallpox was perhaps the most devastating disease for the vernacular peoples, but by no means the only one. According to Cook,

The killer diseases that were not native to the Americas included smallpox, measles, bubonic and pneumonic plague, typhus, and cholera — the last not introduced until the early nineteenth century. All these, except cholera of South Asian origin, existed in various parts of Renaissance Europe, particularly in the larger cities such as Paris, Florence, Genoa, London, and Seville. In the fifteenth century several of these diseases survived in endemic form, persisting just below the surface and exploding into full-fledged epidemics in periods of crisis. (1998, 18)

Of course, these societies also failed to develop preventive methods such as our social distancing or isolation that had been implemented in the European Middle Ages. The point, however, is that America was “virgin soil” (Mann 2005) for the spread of contagious diseases. Contributing to this phenomenon was not only the lack of immunity of the indigenous people, but also the fact that among them, there was no breeding and cohabitation with animals and thus no cross-species transmission of diseases (Stannard 1992, 68). In fact, many domestic animals, such as cows and horses, were imported from Europe and their current presence in America is the result of the Columbian exchange (Mann 2005, 90). According to current research, the first disease transferred from Europe to the Americas was influenza, which arrived with a group of pigs on Columbus’ second voyage to Hispaniola in 1493: “Almost immediately the illness that had come with the fleet of the second expedition spread. The infection passed from sows to horses, and by the time the animals and passengers fled the ship as they disembarked on Hispaniola that November day, virtually all were infected. Illness debilitated the Spaniards, and the highly contagious disease rapidly spread among native peoples as the foreigners fanned out across the island” (Cook 1998, 28–29).

The information that has allowed researchers to reconstruct the invasion of America and the devastating effect of the diseases comes from chronicles and testimonies of the time. The books of *Chilam Balam* belong somehow to this corpus. They are a group of books written in Mayan languages by anonymous scribes, of which several eighteenth and nineteenth century codices found in different Yucatecan towns have been preserved. Each of these texts contains both shared and unique passages. The content of the different books is heterogeneous, but they all refer to the events of the invasion narrated according to Mayan temporality and world view. The following passage is found in *El libro de los libros de Chilam Balam* [The book of books of Chilam Balam], a sort of synthesis of all the books that were known in the mid-twentieth century composed and translated into Spanish by Alfredo Barrera Vásquez and Silvia Rendón:

Enormous work will be the burden of the katun because it will be the beginning of the hangings, the bursting of the fire at the end of the arms of the white people, the ibteeles of the earth who will come with their sabanos and their herds here upon the world, when there falls upon the generation of the Lesser Brothers the rigour of the fight, the rigour of tribute, when the great entrance of tribute comes upon them in the great entrance of Christianity, when the principle of the Seven Sacraments is founded, when much labour begins in the peoples and misery is established in the earth.^7^

There is no mention of disease in this quote, but there are references to other evils that plagued the Maya during the invasion: hangings, the fire at the end of the arms of the whites, the rigor of tribute, and the hard work related to slavery. What I am interested in emphasizing in this passage are the terms *world* and *earth*. As can be seen, from the Mayan perspective, the Spaniards did not belong to the world. Since the “world” was the territory known by the Maya people, the Spaniards were *genuine* “aliens” carrying unknown diseases and misfortunes. The world, we can conclude from here, is always *a* world, it always has a deictic value, and it is that which is “here” where the speaking subjects pronounce their utterance. The Spaniards, therefore, arrived, from somewhere “beyond the world”, and established misery on earth, in what the Maya people considered the *whole* earth.

The result, in demographic terms, is well summarized by Déborah Danowski and Eduardo Viveiros de Castro:

The indigenous population of the continent, larger than that of Europe at the time, may have lost—by means of the combined action of viruses (smallpox in particular being spectacularly lethal), iron, gunpowder, and paper (treaties, papal bulls, royal encomienda concessions, and of course the Bible)—something of the order of 95 percent of its bulk throughout the first one and a half centuries of the Conquest. (2017, 104)^8^

Paralleling these numbers and the current numbers of our COVID-19 pandemic may be revealing: As of 29 March 2023, according to the World Health Organization (2023), there have been 6,887,000 deaths out of a world population of 8,024,780,000 (worldometer 2023), which is less than 0.1%. This is not to minimize our recent experience of dystopia, but to illuminate it from a decentralized or less ethnocentric perspective.

In the Andean region, the invasion and destruction carried out by Europeans is known as Pachakuti. It is a term that is difficult to translate, but which has now entered the vocabulary of post-colonial studies. It implies an abrupt turn in the history, an overturning, and a radical reconfiguration of the previous order. Walter Mignolo writes that “The closest I can get to the limit of Pacha Kuti through the imaginary of modern epistemology (which I cannot avoid) is to translate it as ‘final judgment’” (2011, 158). Enrique Dussel (1995, 109) also refers to the term and its Nahuatl, *tlatzompan*, and Guarani, *mba’e maqua*, equivalents in a sub-chapter of his book *The Invention of the Americas* entitled “The End of the World”.

Scenario 2, therefore, is a closed historical episode. As Ailton Krenak points out,

The simple contagion of the encounter between humans from here and there caused this part of the population to disappear through a phenomenon that was later called an epidemic, a death toll of thousands and thousands of beings. A person who left Europe and landed on a tropical beach left a trail of death where he passed. The individual did not know that he was a walking plague, a bacteriological war in motion, an end of the world; nor did the victims who were contaminated know it. For the people who received that visit and died, the end of the world was in the sixteenth century. (2019, 34, the translation is mine)

“And yet”, Danowski and Viveiros de Castro add, “it just so happens that many of them survived. They carried on in another world, a world of others, their invaders and overlords” (2017, 106). The experience of loss of the world is, however, recorded in indigenous memories; and their current struggles against extractivism are still forms of rejection of the world conceived and imposed by the Western civilizing project, a world based on the accumulation of *capital* (Dussel 1995, 116).

## End

Paralleling the two scenarios is a good procedure to better think through various complexities of our contemporary experience of dystopia. Perhaps because of the unusual transnational media coverage, the spread of the SARS-CoV-2 virus was the first to be perceived as global in scope. The notion of pandemic is, therefore, today more than ever closely related to the concept of the world. However, it could well be argued that, from the perspective of an individual or collective subject, every contagious disease is a global pandemic. The smallpox “epidemics”, which, as such, did not affect Europeans, wiped out at least the Taino *world*, the first indigenous society to be exposed to the European invasion*.* From their perspective, such an “epidemic” was, in addition to a final judgement or a Pachakuti, a global pandemic. Influenza, smallpox, and other diseases, allied with weapons and the Bible, destroyed *their* world.

According to Danowski and Viveiros de Castro 2017, 53), a good starting point is to ask about the place of enunciation, about who says “we”, who this we includes and therefore what is to be understood by the word world and the wor(l)ds.

It is true that the Anthropocene/Capitalocene—depending on the preferred theoretical point of view—has led to a deep and generalized crisis. The aporias of the narrow Western world, which since 1492 has been imposed as a universal paradigm, are now even materially compromising all human beings on the entire planet. However, even the human world is still *a* world. The end of humanity may be, for example, the emancipation or triumph of so many non-human species threatened by human agency. Who says “we” and what that pronominal form includes is therefore a crucial issue. One possible conclusion from the arguments presented in these pages would be that the end of the world made of words, of the Kantian project or of honest liberals, is also the end of *a* world. A world that coined in Europe took the form of the modern ideal that has become universally valid. However, even in the progressive form of radical humanism, it is a world built on a cemetery or on extinct worlds since it is based on the production of otherness and its systematic extermination: that of non-human, more-than-human, and human otherness. Since Western modernity has declared war on nature, on immaterial cosmogonic forces, and on anomalous, marked or dysfunctional subjectivities, the end of *this* world can be interpreted as the end of a project of a clearly destructive character, that is, of the European expansion that began in 1492 and which in the imperial centres became known as modernity, but which in the devastated territories took the name of Pachakuti or tlatzompan. Or it can also be thought of as the end of the radical form taken by the Western civilizing project since the Fall of the Wall and the dismantling of the previous bipolar world. For Timothy Morton (2013), there were other ends of the world: that of 1784, when James Watt invented the steam engine, and that of 1945, when the USA dropped nuclear bombs on Hiroshima and Nagasaki. In any case, for there to be an end of the world, there must first have been a world, even if only as a project. The pandemic, and even the climatic catastrophe or the chain of eschatological phenomena, may be an opportunity for the alterities produced by modernity to conceive another world, different from that of 1492 and 1990. Perhaps, it is necessary and even prudent to sacrifice the human being as agent of the devastation. For the time being, it would not be wrong to take advantage of our experience of the end of *our* world to reflect seriously on our mistakes.
